# Consideration of a Liquid Mutation-Accumulation Experiment to Measure Mutation Rates by Successive Serial Dilution

**DOI:** 10.1093/gbe/evaf049

**Published:** 2025-03-15

**Authors:** Stephan Baehr, Wei-Chin Ho, Samuel Perez, Alyssa Cenzano, Katelyn Hancock, Lea Patrick, Adalyn Brown, Samuel Miller, Michael Lynch

**Affiliations:** Center for Mechanisms of Evolution, Biodesign Institute, Arizona State University, Tempe, AZ, USA; School of Life Sciences, Arizona State University, Tempe, AZ, USA; Center for Mechanisms of Evolution, Biodesign Institute, Arizona State University, Tempe, AZ, USA; Department of Biology, University of Texas, Tyler, TX, USA; Center for Mechanisms of Evolution, Biodesign Institute, Arizona State University, Tempe, AZ, USA; School of Life Sciences, Arizona State University, Tempe, AZ, USA; Center for Mechanisms of Evolution, Biodesign Institute, Arizona State University, Tempe, AZ, USA; Department of Biology, ASU Preparatory Academy Polytechnic High School, Mesa, AZ, USA; Center for Mechanisms of Evolution, Biodesign Institute, Arizona State University, Tempe, AZ, USA; Center for Mechanisms of Evolution, Biodesign Institute, Arizona State University, Tempe, AZ, USA; School of Life Sciences, Arizona State University, Tempe, AZ, USA; Center for Mechanisms of Evolution, Biodesign Institute, Arizona State University, Tempe, AZ, USA; Center for Mechanisms of Evolution, Biodesign Institute, Arizona State University, Tempe, AZ, USA; Center for Mechanisms of Evolution, Biodesign Institute, Arizona State University, Tempe, AZ, USA; School of Life Sciences, Arizona State University, Tempe, AZ, USA

**Keywords:** DNA mutation, mutation accumulation, MMR-*E. coli*, evolution, liquid vs. plate growth

## Abstract

The mutation-accumulation (MA) experiment is a fixture of evolutionary biology, though its execution is laborious. MA experiments typically take between months and years to acquire sufficient mutations to measure DNA mutation rates and mutation spectra. MA experiments for many organisms rely on colony formation on agar plates and repetitive streaking, an environment which at first glance appears somewhat contrived, a poor imitation of actual environmental living conditions. We propose that a fully liquid-phase MA experiment may at times more accurately reflect the environment of an organism. We note also that whereas automation of streaking plates is a daunting prospect, automation of liquid handling, and serial dilution is already commonplace. In principle, this type of MA experiment can be automated so as to reduce the human capital requirements of measuring mutation rates. We demonstrate that a liquid MA recapitulates the mutation rate estimated for MMR-*E. coli* in liquid LB culture vs. plate Lysogeny Broth culture. We detect a modified mutation spectrum with a transition skew of 4.7:1 of A:T→G:C vs. G:C→A:T mutations, highlighting the potential role of tautomerization as a mechanism of DNA mutation.

**Significance**

Some concern has existed regarding the universality of mutation rate estimates generated from studies using agar plates, a contrived environment. The results of this experiment provide an alternative means of running a mutation accumulation (MA) experiment, and suggest that growing bacteria in liquid or on plates does not radically change the amount of selection in an MA experiment, nor the overall estimate of mutation rate. These results support a general applicability of laboratory estimates of mutation rates by MA to our understanding of diverse organisms.

## Introduction

Mutation rates describe the input of heritable variation into an organism. Because the average mutation is deleterious (Eyre-Walker and Keightley 2007), evolution generally pushes DNA mutation rates to the lower limits of natural selection (Lynch et al. 2016). DNA mutation events occur between 1 in 100,000,000 (1/100 million) bases and 1 in 1,000,000,000,000 (1/1 trillion) bases per cell division (Lynch et al. 2016), making their identification within sequenced genomes equivalent to the search for a needle in a rather large haystack. Techniques that amplify the signal of mutation events allow a general means of detection; the fluctuation test, first demonstrated in 1943 (Luria and Delbrück 1943; Foster 2006), uses a selective agent to make certain mutation events stand out, from which mutation rates can be estimated (Lea and Coulson 1949; Zheng 2005; Foster 2006). With the advent of high-throughput sequencing, the mutation-accumulation (MA) experiment (Lynch et al. 2008, 2016), which employs recurrent single-cell bottlenecks typically on some surface like an agar plate, has become the gold standard for the empirical estimation of mutation rates. The analysis of an MA is straightforward, requiring the counting of new mutations arisen over a period of single-cell bottlenecks, and an estimation of the number of cell divisions, so as to come up with the numerator and denominator that comprises a mutation rate.

In microbiology, the MA has generally required growth on agar plates. This requirement reinforces a pervasive bias in microbiology, which typically requires bacteria to be growable on standard plates and in a timely manner, for humans to detect its existence at all. In contrast, metagenomic studies detect enormous diversity and cycling of microbes (Lim et al. 2023; Valles-Colomer et al. 2023), many of which have never been cultured. Some microbes are known to grow quite slowly (Dabkowski et al. 2013; Yoshida et al. 2016; Rodrigues-Oliveira et al. 2023); some do not grow readily on plates at all (Meixner et al. 2022), and others inhabit biological niches that are hard to mimic on plates, for example the human urinary tract, which was once thought to be sterile (Bao et al. 2017). From the output of long-term evolution experiments, to environmental isolates, and medical diagnoses of whether or not someone has a urinary tract infection regardless of what symptoms a patient may report, there is a general acknowledgement agar plates are not universally useful for scoring bacterial presence/growth.

Further specific criticism has been directed to MA experiments. An agar-based or other petri dish likely bears little resemblance to most cellular growth environments, from *E. coli* (Ishii and Sadowsky 2008) to human cells. Oxygen is not a constant presence in many organism environments, though it is almost universally present in MA experiments unless expressly engineered out (Shewaramani et al. 2017). Most cells used for MA experiments grow into colonies of some sort, a growth structure that is nutrient poor and perhaps different from an organism’s natural growth structure, for example in a human gut where we might expect some level of nutrient flow or churn. Starvation has been proposed as a state that may be mutagenic (Taddei et al. 1997; Foster 2007), though typically the time-frames are longer than 24 h, and the experiments have trouble resolving the difference between higher mutation rates or more cell divisions occurring (Katz and Hershberg 2013; Wei et al. 2022). Depending on how a researcher picks and spreads their colonies, edge effects may skew cell division estimates, as some parts of a colony experience more divisions than others (Warren et al. 2019). Though single-cell bottlenecks are expected to overwhelm any but the most stringent selection (lethality), the harmonic mean effective population size is closer to 14 than 1, suggesting that some selection likely occurs during MA experiments. Notably, researchers find compelling evidence for some selection in MA experiments (Mahilkar et al. 2022; Wahl and Agashe 2022); Several authors propose selection is a significant factor in a generic MA experiment, either throughout or in at least the first two weeks (Bosshard et al. 2020). Colony choice instigated by researchers during serial bottlenecking is also at risk of researcher bias, in that humans tend to pick the colonies they can see, rather than just any colony. Humans can also select, consciously or without realizing, a certain colony phenotype, perhaps skewing the distribution of mutations observed in an experiment. The sum of these criticisms is not entirely without merit; there is certainly some selection, positive and purifying, occurring in an MA experiment (Kibota and Lynch 1996; Sung et al. 2015). Regardless, it is difficult to expect mutation-rate estimations are off by more than a factor of 2; for this to be the case, half of all mutations would have to be lethal.

Some of the above concerns of bias may be addressed by an MA experiment with a less structured environment. Researchers have long appreciated the ability of serial dilution to reach countable subsamples of bacterial populations. In principle, some level of dilution can be achieved so that half of all wells inoculated by a dilution contain a viable cell, and half do not. By reaching this level of dilution or lower, relatively high confidence can be maintained that a single viable bacterium was the source of the growth observed in a well 24 h later. Moreover, wild-type *E. coli* can reach carrying capacity within 12–14 h, given a cell division rate of 20–30 min (Tuttle et al. 2021). This suggests that a fixed fitness burden of up to 50% may reasonably be tolerated without affecting our ability to select it. If carrying capacity remains relatively constant, the number of cell divisions should be quite easy to determine relative to a plate MA, which typically develops wildly different colony sizes over time (Lee et al. 2012). The liquid-based single-cell bottleneck eschews the semi-structured agar environment, and reduces the potential risk of artificial selection imposed by researchers. It is also recognizeable that a liquid MA lends itself to automation by liquid handling robots, in contrast to the agar-based MA’s that escape easy conversion to robotic control. If this automation could be achieved, one of the most laborious aspects of obtaining mutation rates by an MA experiment could be made more tolerable. We did not automate our liquid MA, but the point remains enticing for future work.

**Fig. 1.**
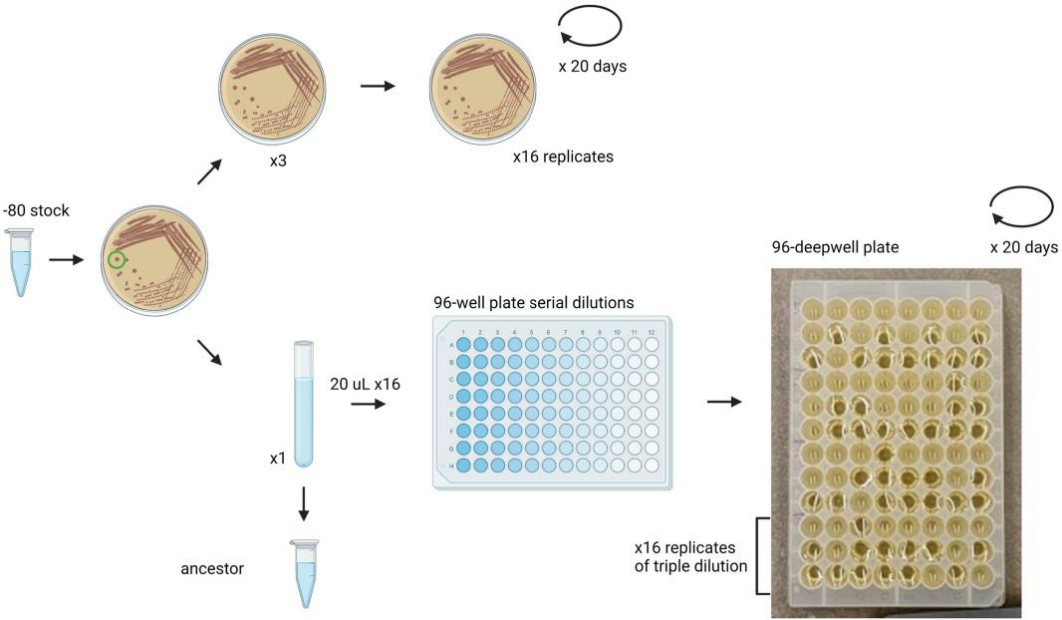
Experimental design. From a single-colony streaked on a plate, liquid and plate MA’s were started in tandem. The plate MA used standard methods. The liquid MA begins with a serial dilution to the point of getting 0 or 1 cells in a well. Three different serial dilutions were used to ensure sufficient dilution.

To compare and contrast the output of MA experiments run by liquid serial dilution or by standard agar plates, we chose to use a well-studied *E. coli* strain, MutL- also known as mismatch-repair deficient (MMR-), K12, MG1655, originating from the lab of Pat Foster. This train has already been characterized by a standard MA experiment, at least twice (Lee et al. 2012; Wei et al. 2022). This strain is particularly useful because it features a distinct mutation spectrum, as well as an elevated mutation rate, at least 100-fold higher than WT. The elevated mutation rate allows for a temporally shorter MA with relatively few samples to yield an abundance of mutations, along with measureable phenotypes. We ran a plate and a liquid MA in tandem from the same starting single colony for 20 days (Fig. 1), and afterward sequenced the DNA. We find a modest difference in the mutation spectrum of *E. coli*, but moreso recognize the relative invariance of mutation-rates estimated from the experiment between growth types, $3.10\times 10^{-8}$ vs. $2.80\times 10^{-8}$ base-pair substitutions (BPS)/site/generation for plate and liquid MA’s, respectively. These data suggest the liquid MA is a compelling option for identifying the mutation rates for micro-organisms which do not grow well on agar surfaces.

## Results

**Fig. 2.**
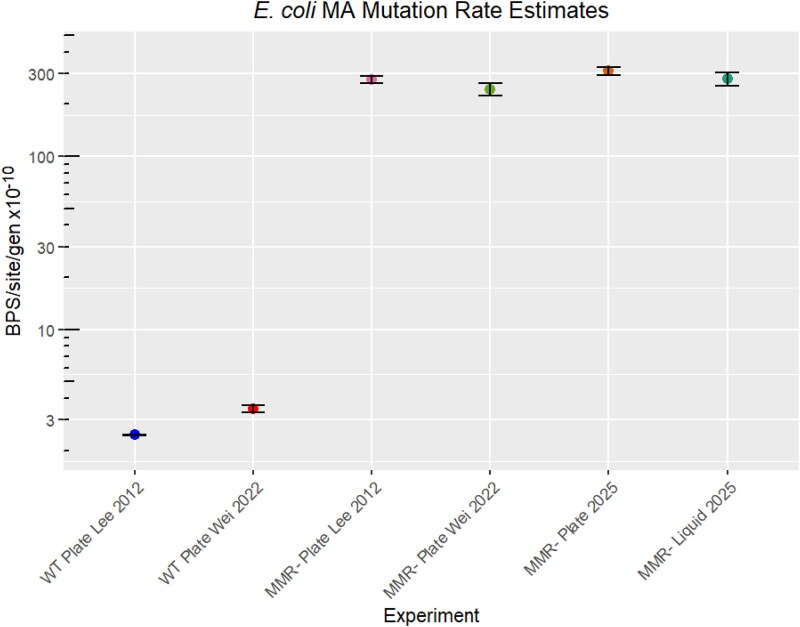
Mutation rate estimation. Multiple estimates of the *E. coli* MMR-mutation rate are consistent over time. The liquid and plate experiments of this report produced mutation rate estimates more similar to each other than the difference between plate MA experiments over time. Error bars reflect the standard error of the mean.

The tandem MA experiments run in liquid and upon plates of otherwise identical nutrient content produced highly similar estimates of mutation rate per site per generation, and are in general agreement with previous MA experiments (Fig. 2). This resulted in similar mutation burdens over the course of the experiment (Table 1). The modest difference in the mutation rate is explained by the liquid MA experiencing slightly more cell divisions per day. Specifically, the volume of the 96-deepwell plates allowed for the liquid MA lines to experience 10% more cell divisions (29.9 vs. 26.9 mean cell divisions per line per day). Also in concurrence with previous MA experiments (Lee et al. 2012), we find the indel rate to be reduced relative to base-pair substitution rates (Table 1). This is a general trend among *E. coli* and most other organisms (Sung et al. 2016; Lynch et al. 2023).

**Table 1.** Summary statistics for the liquid and plate MA experiments. SEM: standard error of the mean

Treatment	Sample size	Mean BPS per line (SEM)	Mean INDEL per line	Mean generations per transfer	Mutation BPS/site/gen $\times 10^{-8}$ (SEM)	indel/site /gen $\times 10^{-8}$ (SEM)
Liquid	16	76.8 (± 6.32)	17.8 (± 1.44)	29.9	2.80 (± 0.24)	0.65 (± 0.053)
Plate	15	76.7 (± 4.28)	12.3 (± 0.78)	26.9	3.10 (± 0.17)	0.49 (± 0.031)

**Fig. 3.**
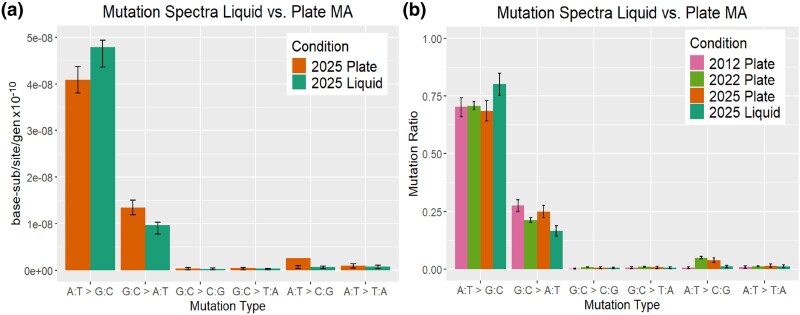
Mutation spectra of MMR-*E. coli*. a) Across MA experiments the mutation spectrum ratios of MMR-*E. coli* mutations holds relatively constant, particularly in the case of the plate MA. b) Mutation rates rather than mutation ratios. In this graph, the mutations from hypermutator line P35 are omitted. Error bars reflect poisson 95% CIs.

The mutation spectra are similar to previous estimates from 2012 and 2022 (Fig. 3a), although a $\chi^{2}$ test detected a significant difference between liquid and plate spectra (Fig. 3b) (P=8.8×e−8), driven by both a reduction in G:C→A:T transitions and an increase in A:T→G:C transitions (Table 2). Though the difference by ratio is modest in appearance, the net effect over 20 days yields a difference of 74 G:C→A:T mutations across summed across the 16 lines (Table 2), a 37% increase in occurrence in the plate vs. liquid MA experiments.

**Table 2.** Raw data of the mutation spectrum

Mutation type	L Total count	L Frequency	P Total count	P Frequency
A:T→G:C	995	0.81	806	0.71
G:C→A:T	198	0.16	272	0.236
A:T→C:G	12	0.010	13	0.011
A:T→T:A	15	0.012	18	0.016
G:C→T:A	5	0.004	6	0.005
G:C→C:G	4	0.003	7	0.006

**Fig. 4.**
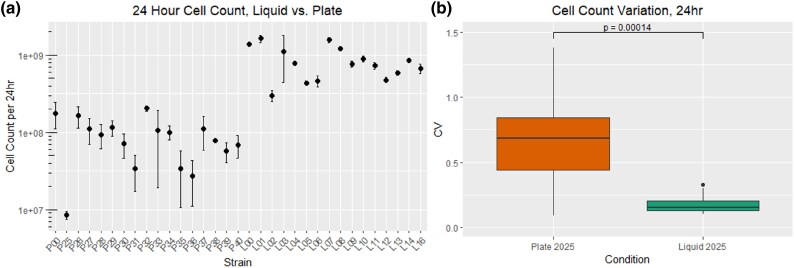
Cell counts per day, MMR-*E. coli*. a) CFU estimate of the number of cells present in a culture after 24 h of growth, from resuspended colonies or from 1 mL liquid cultures for the plate and liquid MA’s, respectively. b) Coefficient of Variation (CV) summed over the two MA’s, indicating that there is a greater CV and distribution of CV in the plate MA than the liquid MA. Statistical significance was calculated the Mann–Whitney *U* test, as the distribution of the liquid MA CV estimates were not normally distributed.

The liquid MA experienced roughly three additional mutations per day (29.9 vs. 26.9, Pvalue=7.662×e−11, t-test), a consequence of the 1 mL growth volume allotted per well in the 96-deepwell plate. We note that a difference in the number of cell divisions is not only in count per day but also in variation per colony, which is readily identified in a comparison of the standard error of the mean in the estimates of number of cells per day (Fig. 4). The increased variance, readily discernable by eye in Fig. 4a, error bars liquid samples vs. plate samples, can be compared by calculating the coefficient of variation (CV), which normalizes the standard deviation of a sample by its mean value. The difference in CV is statistically significant (Fig. 4b, Pvalue=0.00014, Mann–Whitney *U* test), and is readily explained by colony dynamics. Specifically, when colonies are close to one another they experience nutrient competition and grow less quickly. Random differences in spacing between colonies leads to variation in colony size, and thus increased variation in the total number of cell divisions experienced in a day relative to liquid cultures. It is also true that colonies shrink on average over the course over a MA experiment, as mutations accumulate and fitness degrades. This shrinking colony size is readily seen in measurements of fitness, for example in plate line 25 (Fig. 4a).

**Fig. 5.**
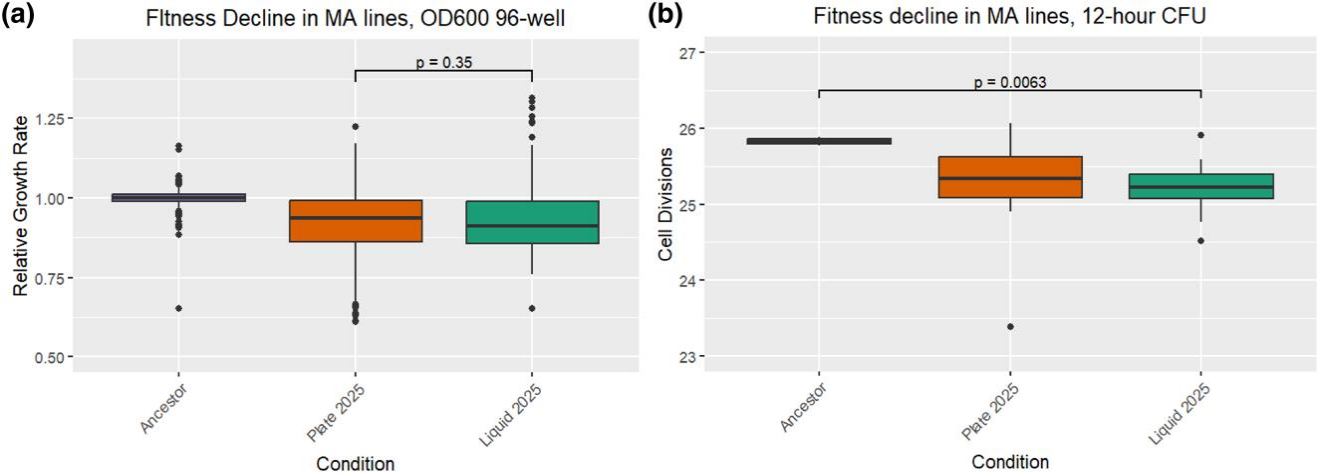
Fitness declines in liquid and plate MA of MMR-*E. coli*. a) Relative decrease in fitness after 20 days of MA in liquid and plate conditions, after 12 h of growth, as measured by colony forming units (CFU). b) Relative decrease in fitness after 20 days of MA in liquid and plate conditions, after 24 h of growth, as measured by an OD600 96-well plate reader.

One hallmark of a successful MA experiment is a decline in mean fitness of the lines (Kibota and Lynch 1996). To ensure our MA ran as anticipated, we measured fitness through use of a 96-well plate reader, which determines changes in optical-density over time. In particular, the maximum slope of optical-density change in the first 8 h of the experiment is sufficient to provide a useful proxy for maximum cell growth rate (Fig. 5a), as has been employed elsewhere. We additionally verified the results from the 96-well plate reader by measuring CFU’s of the 12 h time-point (Fig. 5b). Based on the growth rate change, we calculate the average fitness cost per mutation to be −0.00085 (SEM 0.00028) and −0.00079 (SEM 0.00027), for liquid and plate samples, respectively. In this calculation, we use the sum burden of indels and base substitutions. We note, as have others (Baer et al. 2005), the relatively high variance in the fitness cost per mutation per line; this is likely due to rare alleles of strongly deleterious or positive effect. We note that the true baseline mean fitness cost per mutation for MMR-*E. coli* in LB culture is assuredly more deleterious, given our present report (below) of evidence of purifying selection, particularly among indels, in MA experiments. This is in line with prior reports (Wahl and Agashe 2022).

In support of previous experimental results, the degree of selection within the MA was found to be significant though modest in nature. If mutations accumulate in an entirely neutral process, we would expect a ratio of 5.74 coding mutations per every 1 noncoding mutation, a reflection of the ratio of coding to noncoding DNA in *E. coli* MG1655 (Lee et al. 2012), or $3.95\times 10^{6}$ nucleotides coding to $6.88\times 10^{5}$ noncoding nucleotides. The occurrence of indels and base-subs at intergenic (between genes) or intragenic (within genes) nucleotide site is expected to be somewhat lower than the neutral expectation, in account of the advent of lethal mutations, with a stronger effect on indels due to their more disruptive character. We find that the ratio of intergenic/intragenic indels is 2.68:1 in the liquid MA and 2.54 in the plate MA, significantly different from the expected ratio of 5.74:1 (1.15× e-28, $\chi^{2}$). Further, for base-subs we find the intragenic/intergenic ratio to be 9.91:1 (*P*-value 2.62×e-74*χ*-square) and 7.71:1, for liquid and plate MA’s, respectively. These results indicate the evidence of purifying selection in the case of indels (fewer indels in coding sequences relative to null expectation), and positive selection for base substitutions in coding sequences. These trends are in line with recent findings of selection within MA experiments (Mahilkar et al. 2022; Wahl and Agashe 2022), particularly when more cell divisions between bottlenecks are built into the experimental design, as was the case of the liquid MA.

Within coding nucleotides, the expected ratio of nonsynonymous to synonymous mutations is 3.10, corresponding to 29,42,979 effectively nonsynonymous sites to 9,47,643 effectively synonymous sites. Compared with the expected ratio, we detect a ratio of 2.13 in the liquid MA and 2.07 for the Plate MA. Both deviate from the null expectation ($\chi^{2}P=2.27\times 10^{-21}$), but are not significantly different from one another. These results are nearly identical to those reported in 2012 (Lee et al. 2012). The difference in ratios suggest an excess of synonymous mutations or a dearth of nonsynonymous mutations compared with the null expectation in MMR-*E. coli*. This is additionally in line with recent commentary on bacterial MA experiments (Mahilkar et al. 2022; Wahl and Agashe 2022).

With regard to the comparison, the Liquid MA appears to yield comparable results to the standard plate MA. It is possible that, with selection likely reducing the ratio of nonsynonymous mutations from 3.1:1 to 2.1:1, the true mutation rate of MMR-*E. coli* may be roughly a third higher than is reported here and in previous experiments, or $3.8\times 10^{-8}$ base pair substitutions per site per generation.

## Discussion

In summary, the liquid MA largely recapitulates the findings of a plate MA for MMR-*E. coli*, with several modest differences. This result supports the perspective that MA experiments, even those which have been run on agar plates, provide reasonable estimates of organism mutation rates. It would appear that *E. coli* is not significantly perturbed in mutation rate by growing either in liquid or on plate colonies, over the course of 24 h. Despite some fraction of the cells being nutrient deprived for 6–14 h (Warren et al. 2019), *E. coli* appears evolved to manage this environmental challenge with no significant change to its genome-wide mutation rate. Given the expectation that bacteria routinely encounter nutrient deprivation for hours, or perhaps even days or months in their evolutionary past, this result is not exceptional.

A primary finding of this research is that the liquid MA, single-cell bottlenecking by serial dilution works equivalently to colonies on plates, despite differences in bacterial growth environment. It must be noted that by human hand, the task of streaking 16 samples on plates takes no more than 16 min’s work even for the most unfamiliar. The liquid MA turns this into a task of about 90 min, a poor incentive for anyone considering replicating the work presented in this article. However, the potential strengths of the liquid MA will lie in its scalability and the promise of automation, which could in principle require no more than picking which well a researcher would like to load per sample into a robot for serial dilution. In the hands of a robot, we expect that contamination would be somewhat less of a risk while running MA experiments, though we note that proper biosafety cabinet technique by hand also results in a relatively low risk of contamination. Given that some organisms do not grow well on agar plates, this protocol may also provide some researchers with an avenue to pursue questions of variation in mutation rate which have previously been intractable.

We note the overall resilience of the standard MA in the face of some criticisms to the technique over the years. Despite fear of induced selection or bias on behalf of the colony picker, our cursory examination of MA results between liquid and plate demonstrate the reliability of both protocols. We do not detect a large difference in mutation rate, in mean fitness cost per mutation, or in DN/DS ratio. One minor benefit of running a liquid MA is a less variant number of cell divisions per day per line. This may result in a more accurate estimation of mutation rates, though if true the effect is modest, if present at all: we detect a difference of 2% between the estimates of mutation rate between liquid and plate MA’s. In summary, the current evidence suggests that MA experiments in labs are fair proxies of the organism’s overall mutation rate.

The statistically significant, though modest difference in mutation spectrum is curious, and for this we have no immediate explanation. Before putting much weight in speculation, we would like to see the result reproduced. We note that some plate MMR-MA lines from the Foster lab have produced mutation spectra of similar (Foster et al. 2018) skew to those reported here. With this preface, we note that the liquid mutation rate is skewed even more strongly to A:T→G:C than the previous results of plate MA experiments. The most parsimonious mechanism for this mutation spectrum of transitions is base-pair tautomerization, as proposed by Watson and Crick in 1953 (Watson and Crick 1953) and iterated upon since (Slocombe et al. 2021). Watson and Crick noted that the temporary shift of hydrogen atoms to neighboring atoms might cause some level of nucleotide mispairing. They hypothesized that the A:T→G:C mutation ought to be more frequent than the G:C→A:T, because the former requires a single hydrogen atom shift, while the latter requires two hydrogen atom shifts. It is unclear if or why the medium of growth would alter the ratio of tautomerization, though it is known that the spent culture of *E. coli* significantly changes in pH (increase to pH of 9) and chemical composition (Sezonov et al. 2007; Behringer et al. 2018) which also affects gene expression profiles. Further, the decreased G:C→A:T rate may be partly explained by the *E. coli* of the liquid MA being exposed to less oxidative stress resulting in fewer damaged guanosine residues, a base prone to oxidative damage (Kino et al. 2017; Hahm et al. 2022). However, growth of wild-type *E. coli* in the complete absence of oxygen mildly increased mutation rates, on the order of 2-fold, with no reduction in mutations to guanosines (Shewaramani et al. 2017). Regardless, the recurrent theme of MMR-*E. coli* and its derived strains are that the mutation spectrum is skewed in favor of transitions, as expected (Lee et al. 2012; Wei et al. 2022). The data of this work and prior MMR-growth experiments lend credence to the idea that tautomerization, if rare, is likely biologically relevant, such that life evolves DNA-repair pathways to manage its occurrence. We note that the degree of the imbalance in mutation types is potentially quite useful in the verification of novel sequencing technologies seeking to detect DNA mutations, to ensure that they are working properly.

A retrospective of the experiment suggests potential avenues for improved efficiency. The experimental design was strongly influenced by a desire for relative surety of obtaining single-cell bottlenecks. Alternative strategies exist, for example one in which serial dilutions are performed by doing 1:2 dilutions from a starting $10^{-6}$ dilution. Within eight serial dilutions, the dilution should be complete, and yield wells which have no growth inside them. A researcher would then simply select the last serial dilution with a grown bacterial culture. This strategy would yield strong bottlenecks, which would allow a fair approximation of mutation rate with higher throughput. Instead of 4 samples per 96-deepwell plate, the yield would become 12. The dilution strategy of the deepwell plate could also make use of a multichannel pipettor; the protocol described in this experiment did not, when inoculating each row of serial dilution. There is some risk of increased selection in the experiment, but the protocol would be perhaps 2–3× faster with a 3× increase in yield.

## Methods

Frozen stocks of *E. coli* MMR- were thawed and streaked upon an LB plate (LB Agar, Miller). 24 h later, a single colony was chosen, from which one 10 mL liquid LB tube (LB Broth, Miller) was inoculated while 4 additional LB plates were streaked. From the four agar plates, 40 colonies were chosen to be the set of 40 MA lines, and comprised the first day of the plate MA. For each of these 40 lines, per day, every 24 h, a single colony was selected and streaked on half of an LB plate, with two or three streaks to obtain single colonies. The first streak was a toothpick line (autoclaved toothpicks), and one or two consecutive streaks thereafter came from autoclaved wooden sticks. The last colony of a streak was always chosen for transfer, unless forced by poor streaking to pick the last convincing single colony. Plates were loaded into a 37°C incubator and grown overnight for 24 h, ± 2 h, before streaking was repeated on new LB agar plates the next day.

From the 10 mL liquid LB tube, first an ancestor line frozen stock was made and then 16 wells of 20 μL were taken and run through serial dilution (Fig. 1). Briefly, *E. coli* were serially diluted to the −5 and −6 wells by standard methods and multichannel pipettors in 180 μL 1× PBS in 96-well plates (supplementary Fig. 1, Supplementary Material online). Then, 100 μL of the −5 was added to 900 μL of 1× PBS to create a “−7” dilution of 1 mL per sample, and the same was done to the −6 well to create a “−8” dilution of 1 mL per sample. From the −7 dilution, 20 μL was inoculated to a single row of a 96-deepwell plate holding 950 μL of liquid LB, 8 wells. From the −8 dilution, a second row for the same sample was loaded with 50 μL, and for a 3rd row the −8 dilution was again used to inoculate 20 μL. This results in an order of magnitude scale of dilution in the 96-deepwell plate, where each liquid sample comprises now 24 replicates of varying serial dilution. On a 96-deepwell plate four samples were arrayed, for a total of 4 96-deepwell plates, or 16 replicates, per transfer day. After 24 h, some of the serial dilution wells have turbid growth, while others remain clear. At the lower serial dilutions, a majority of the wells should be clear. As a rule, the turbid well from the lowest serial dilution would be chosen for the next round of serial dilution. In the event of a tie, the one on the left-hand side was chosen. After 15 days, frozen stocks were made of both the liquid and the plate MA. After noticing an apparent decrease in carrying capacity in one of the liquid MA lines, the experiment was stopped at 20 days for characterization and sequencing. Based on the previous MA experiment (Lee et al. 2012), we expected 20 days would result in a sufficient number of mutations to analyze the data.

After the MA’s were complete and frozen stocks were obtained, DNA was extracted by first growing the frozen stocks overnight in 10 mL tubes and then running the Promega Wizard DNA Extraction Kit, with minor modifications immaterial to results save by increasing yield to >1μg per sample. Genomic DNA was quantified, and submitted to the Beijing Genomics Institute for their in house library prep and DNB-sequencing.

Raw sequence reads were analyzed by fastqc to confirm a successful sequencing run. Reads were then filtered with Trimmomatic to remove adapter sequences. Reads were then aligned to the *E. coli* K12 reference genome (https://www.ncbi.nlm.nih.gov/nuccore/U00096.2) with BWA, the Burrows-Wheeler Aligner. After alignment and conversion to bam files by SamTools, the mutation caller GATK2 generated VCF files of novel mutations when comparing the ancestor to the evolved lines. Output VCF files were additionally annotated by SnpEff to determine Dn/Ds and Genic/Intergenic ratios. We graphed the results of the mutation rate and mutation spectrum with ggplot2 in R. Error bars for the mutation spectrum reflect the poisson 95% CIs, similar to previous studies, which were calculated using the Epitools package for R with pois.approx.

To estimate the number of cell divisions that occurred over the course of the 20-day experiment, the liquid and plate MAs were handled differently. For the plate MA, 20-day frozen stocks and the ancestor were thawed and grown in LB overnight in 10 mL tubes. This liquid was then streaked on LB plates. After 24 h, the colonies were resuspended in LB and serially diluted to the −6 plate, and plated to count colony forming units (CFU’s) per mL. An average of the entire set of 15 plate MA samples was used to calculate mean number of cell divisions per day at day 20, the ancestor calculated mean number of cell divisions per day at day 1. The average between the day 1 and mean of day 20 samples was used as an estimate for number of cell divisions per line.

To estimate cell divisions from the liquid MA, 20-day frozen stocks and the ancestor were thawed and grown overnight in LB in 10 mL tubes. After 24 h, 20 μL aliquots were taken from the overnight cultures and serially diluted down to single cell bottlenecks, before being grown up in 96-deepwell plates, in a method identical to the MA serial dilution. After an additional 24 h, four turbid replicates were chosen for serial dilution to count CFUs. From this we calculated the number of cell divisions per day, at day 1 and day 20. The mean of day 20 cell division estimates, and the average between the day 1 number of cell divisions and mean of day 20 lines was used as an estimate for average total number of cell divisions during the experiment.

To phenotype the lines for fitness and carrying capacity using a plate reader, a 96-well plate was used. Two lines and an ancestor were thawed out per day and grown overnight. 100 μL of overnight culture was then added to 10 mL of LB in 15 mL tubes. These 15 mL tubes were inverted 15 times to mix. The mixtures were then loaded onto a 96-well plate, 200 μL per plate, staggered such that rows 1, 4, 7, and 10 received the ancestor, 2, 5, 8, 11 received MA line 1, and 3, 6, 9, 12 received MA Line 2. the 96-well plate was double-sealed with parafilm and loaded into a plate reader for 24 h of optical-density analysis, at 37°C, with readings taken at a wavelength of 600 nm (OD600). Growth rate was obtained by calculating the largest slope of growth between 2 and 10 h, when averaged across 5 time points. Carrying capacity was calculated at average optical-density at 12 h, averaged over 5 time points. Data were normalized per day in comparison to the ancestor, and then the relative fitness and relative “carrying capacity” were graphed in R via a box-and-whisker plot. Dots on the plot are extreme outliers of the box-and-whisker plot. Outliers arose with a low frequency, due to uncommon tears in a double-parafilm layer deployed to prevent evaporation. Small tears resulted in local evaporation, which randomly affected all lines. No wells were discarded in the measurement of relative fitness, regardless of their outlier state. The outliers were less than 10% of any given sample, and thus we consider any impact on our estimates of fitness to be a second-order effect; and even so, that effect was evenly distributed across the lines, from which a relative fitness performance was measured.

To phenotype the lines for carrying capacity after 12 h by CFU, all lines were thawed in 10 mL tubes of LB overnight. The lines were then diluted in quadruplicate to 1/100th concentration in a 96-well plate, by adding 2 μL of mixed overnight culture to 198 μL of fresh LB. These plates were then sealed with parafilm in 2 layers and loaded into a 37 degree incushaker shaking at 200RPM, secured by magnets, for 12 h. After 12 h, the *E. coli* were serial diluted to the −7 plate and CFUs were obtained by spreading 100 μL on LB plates. Data were processed by collecting CFU estimates, running t tests, and graphing the different CFU averages with standard error of the mean in R.

## Supplementary Material

evaf049_Supplementary_Data

## Data Availability

The data generated by this study are available at the SRA With bioproject number PRJNA1096062. Supplementary files, Supplementary Material online containing all the mutations called can be found attached to this manuscript. The command line, R, and python code used to analyze and interpret the data can be found at https://github.com/biosbaehr/liquid_vs_plate_MA

## References

[evaf049-B1] Baer CF, Shaw F, Steding C, Baumgartner M, Hawkins A, Houppert A, Mason N, Reed M, Simonelic K, Woodard W, et al Comparative evolutionary genetics of spontaneous mutations affecting fitness in rhabditid nematodes. Proc Natl Acad Sci U S A. 2005:102(16):5785–5790. 10.1073/pnas.0406056102. 15809433 PMC556281

[evaf049-B2] Bao Y, Al KF, Chanyi RM, Whiteside S, Dewar M, Razvi H, Reid G, Burton JP. Questions and challenges associated with studying the microbiome of the urinary tract. Ann Transl Med. 2017:5:33. 10.21037/atm.2016.12.14. 28217698 PMC5300849

[evaf049-B3] Behringer MG, Choi BI, Miller SF, Doak TG, Karty JA, Guo W, Lynch M. *Escherichia coli* cultures maintain stable subpopulation structure during long-term evolution. Proc Natl Acad Sci U S A. 2018:115(20):E4642–E4650. 10.1073/pnas.1708371115. 29712844 PMC5960275

[evaf049-B4] Bosshard L, Peischl S, Ackermann M, Excoffier L. Dissection of the mutation accumulation process during bacterial range expansions. BMC Genomics. 2020:21(1):253. 10.1186/s12864-020-6676-z. 32293258 PMC7092555

[evaf049-B5] Dabkowski J, Dodds P, Hughes K, Bush M. A persistent, symptomatic urinary tract infection with multiple “negative” urine cultures. Conn Med. 2013:77(1):27–29. 23427370

[evaf049-B6] Eyre-Walker A, Keightley PD. The distribution of fitness effects of new mutations. Nat Rev Genet. 2007:8(8):610–618. 10.1038/nrg2146. 17637733

[evaf049-B7] Foster PL . Methods for determining spontaneous mutation rates. In: DNA Repair, Part B, of Methods in Enzymology. Vol. 409. Academic Press; 2006. p. 195–213. 10.1016/S0076-6879(05)09012-9 PMC2041832 16793403

[evaf049-B8] Foster PL . Stress-induced mutagenesis in bacteria. Crit Rev Biochem Mol Biol. 2007:42(5):373–397. 10.1080/10409230701648494. 17917873 PMC2747772

[evaf049-B9] Foster PL, Niccum BA, Popodi E, Townes JP, Lee H, MohammedIsmail W, Tang H. Determinants of base-pair substitution patterns revealed by whole-genome sequencing of DNA mismatch repair defective *Escherichia coli*. Genetics. 2018:209(4):1029–1042. 10.1534/genetics.118.301237. 29907647 PMC6063221

[evaf049-B10] Hahm JY, Park J, Jang E-S, Chi SW. 8-Oxoguanine: from oxidative damage to epigenetic and epitranscriptional modification. Exp Mol Med. 2022:54(10):1626–1642. 10.1038/s12276-022-00822-z. 36266447 PMC9636213

[evaf049-B11] Ishii S, Sadowsky MJ. *Escherichia coli* in the environment: implications for water quality and human health. Microbes Environ. 2008:23(2):101–108. 10.1264/jsme2.23.101. 21558695

[evaf049-B12] Katz S, Hershberg R. Elevated mutagenesis does not explain the increased frequency of antibiotic resistant mutants in starved aging colonies. PLoS Genet. 2013:9(11):1–10. 10.1371/journal.pgen.1003968. PMC3828146 24244205

[evaf049-B13] Kibota TT, Lynch M. Estimate of the genomic mutation rate deleterious to overall fitness in E. coli. Nature. 1996:381(6584):694–696. 10.1038/381694a0. 8649513

[evaf049-B14] Kino K, Hirao-Suzuki M, Morikawa M, Sakaga A, Miyazawa H. Generation, repair and replication of guanine oxidation products. Genes Environ. 2017:39(1):21. 10.1186/s41021-017-0081-0. 28781714 PMC5537945

[evaf049-B15] Lea DE, Coulson CA. The distribution of the numbers of mutants in bacterial populations. J Genet. 1949:49(3):264–285. 10.1007/BF02986080. 24536673

[evaf049-B16] Lee H, Popodi E, Tang H, Foster PL. Rate and molecular spectrum of spontaneous mutations in the bacterium Escherichia coli as determined by whole-genome sequencing. Proc Natl Acad Sci U S A. 2012:109(41):E2774–2783. 10.1073/pnas.1210309109. 22991466 PMC3478608

[evaf049-B17] Lim JJ, Diener C, Wilson J, Valenzuela JJ, Baliga NS, Gibbons SM. Growth phase estimation for abundant bacterial populations sampled longitudinally from human stool metagenomes. Nat Commun. 2023:14(1):5682. 10.1038/s41467-023-41424-1. 37709733 PMC10502120

[evaf049-B18] Luria SE, Delbrück M. Mutations of bacteria from virus sensitivity to virus resistance. Genetics. 1943:28(6):491–511. 10.1093/genetics/28.6.491. 17247100 PMC1209226

[evaf049-B19] Lynch M, Ackerman MS, Gout J-F, Long H, Sung W, Thomas WK, Foster PL. Genetic drift, selection and the evolution of the mutation rate. Nat Rev Genet. 2016:17(11):704–714. 10.1038/nrg.2016.104. 27739533

[evaf049-B20] Lynch M, Ali F, Lin T, Wang Y, Ni J, Long H. The divergence of mutation rates and spectra across the tree of life. EMBO Rep. 2023:24(10):e57561. 10.15252/embr.202357561. 37615267 PMC10561183

[evaf049-B21] Lynch M, Sung W, Morris K, Coffey N, Landry CR, Dopman EB, Dickinson WJ, Okamoto K, Kulkarni S, Hartl DL, et al A genome-wide view of the spectrum of spontaneous mutations in yeast. Proc Natl Acad Sci U S A. 2008:105(27):9272–9277. 10.1073/pnas.0803466105. 18583475 PMC2453693

[evaf049-B22] Mahilkar A, Raj N, Kemkar S, Saini S. Selection in a growing colony biases results of mutation accumulation experiments. Sci Rep. 2022:12(1):15470. 10.1038/s41598-022-19928-5. 36104390 PMC9475022

[evaf049-B23] Meixner K, Daffert C, Bauer L, Drosg B, Fritz I. PHB producing cyanobacteria found in the neighborhood-their isolation, purification and performance testing. Bioengineering (Basel). 2022:9(4):178. 10.3390/bioengineering9040178. 35447738 PMC9030849

[evaf049-B24] Rodrigues-Oliveira T, Wollweber F, Ponce-Toledo RI, Xu J, R. Rittmann SK-M, Klingl A, Pilhofer M, Schleper C. Actin cytoskeleton and complex cell architecture in an Asgard archaeon. Nature. 2023:613(7943):332–339. 10.1038/s41586-022-05550-y. 36544020 PMC9834061

[evaf049-B25] Sezonov G, Joseleau-Petit D, D’Ari R. Escherichia coli physiology in Luria-Bertani broth. J Bacteriol. 2007:189(23):8746–8749. 10.1128/JB.01368-07. 17905994 PMC2168924

[evaf049-B26] Shewaramani S, Finn TJ, Leahy SC, Kassen R, Rainey PB, Moon CD. Anaerobically grown Escherichia coli has an enhanced mutation rate and distinct mutational spectra. PLoS Genet. 2017:13(1):1–22. 10.1371/journal.pgen.1006570. PMC5289635 28103245

[evaf049-B27] Slocombe L, Al-Khalili JS, Sacchi M. Quantum and classical effects in DNA point mutations: Watson-Crick tautomerism in AT and GC base pairs. Phys Chem Chem Phys. 2021:23(7):4141–4150. 10.1039/D0CP05781A. 33533770

[evaf049-B28] Sung W, Ackerman MS, Dillon MM, Platt TG, Fuqua C, Cooper VS, Lynch M. Evolution of the insertion-deletion mutation rate across the tree of life. G3 (Bethesda). 2016:6(8):2583–2591. 10.1534/g3.116.030890. 27317782 PMC4978911

[evaf049-B29] Sung W, Ackerman MS, Gout J-F, Miller SF, Williams E, Foster PL, Lynch M. Asymmetric context-dependent mutation patterns revealed through mutation–accumulation experiments. Mol Biol Evol. 2015:32(7):1672–1683. https://academic.oup.com/mbe/article-pdf/32/7/1672/13166567/msv055.pdf. 10.1093/molbev/msv055. 25750180 PMC4476155

[evaf049-B30] Taddei F, Halliday JA, Matic I, Radman M. Genetic analysis of mutagenesis in aging Escherichia coli colonies. Mol Gen Genet. 1997:256(3):277–281. 10.1007/s004380050570. 9393452

[evaf049-B31] Tuttle AR, Trahan ND, Son MS. Growth and maintenance of Escherichia coli laboratory strains. Curr Protoc. 2021:1(1):e20. 10.1002/cpz1.v1.1. 33484484 PMC8006063

[evaf049-B32] Valles-Colomer M, Blanco-Míguez A, Manghi P, Asnicar F, Dubois L, Golzato D, Armanini F, Cumbo F, Huang KD, Manara S, et al The person-to-person transmission landscape of the gut and oral microbiomes. Nature. 2023:614(7946):125–135. 10.1038/s41586-022-05620-1. 36653448 PMC9892008

[evaf049-B33] Wahl LM, Agashe D. Selection bias in mutation accumulation. Evolution. 2022:76(3):528–540. 10.1111/evo.v76.3. 34989408

[evaf049-B34] Warren MR, Sun H, Yan Y, Cremer J, Li B, Hwa T. Spatiotemporal establishment of dense bacterial colonies growing on hard agar. Elife. 2019:8:e41093. 10.7554/eLife.41093. 30855227 PMC6411370

[evaf049-B35] Watson JD, Crick FH. The structure of DNA. Cold Spring Harb Symp Quant Biol. 1953:18(0):123–131. 10.1101/SQB.1953.018.01.020. 13168976

[evaf049-B36] Wei W, Ho W-C, Behringer MG, Miller SF, Bcharah G, Lynch M. Rapid evolution of mutation rate and spectrum in response to environmental and population-genetic challenges. Nat Commun. 2022:13(1):4752. 10.1038/s41467-022-32353-6. 35963846 PMC9376063

[evaf049-B37] Yoshida S, Hiraga K, Takehana T, Taniguchi I, Yamaji H, Maeda Y, Toyohara K, Miyamoto K, Kimura Y, Oda K. A bacterium that degrades and assimilates poly(ethylene terephthalate). Science. 2016:351(6278):1196–1199. 10.1126/science.aad6359. 26965627

[evaf049-B38] Zheng Q . New algorithms for Luria–Delbrück fluctuation analysis. Math Biosci. 2005:196(2):198–214. 10.1016/j.mbs.2005.03.011. 15950991

