# Supplementary figures and images for: Consideration of a Liquid Mutation-Accumulation Experiment to Measure Mutation Rates by Successive Serial Dilution
Source: Genome Biol Evol. 2025 Mar 15;17(4):evaf049. doi: 10.1093/gbe/evaf049 (PMC11973482; doi:10.1093/gbe/evaf049)

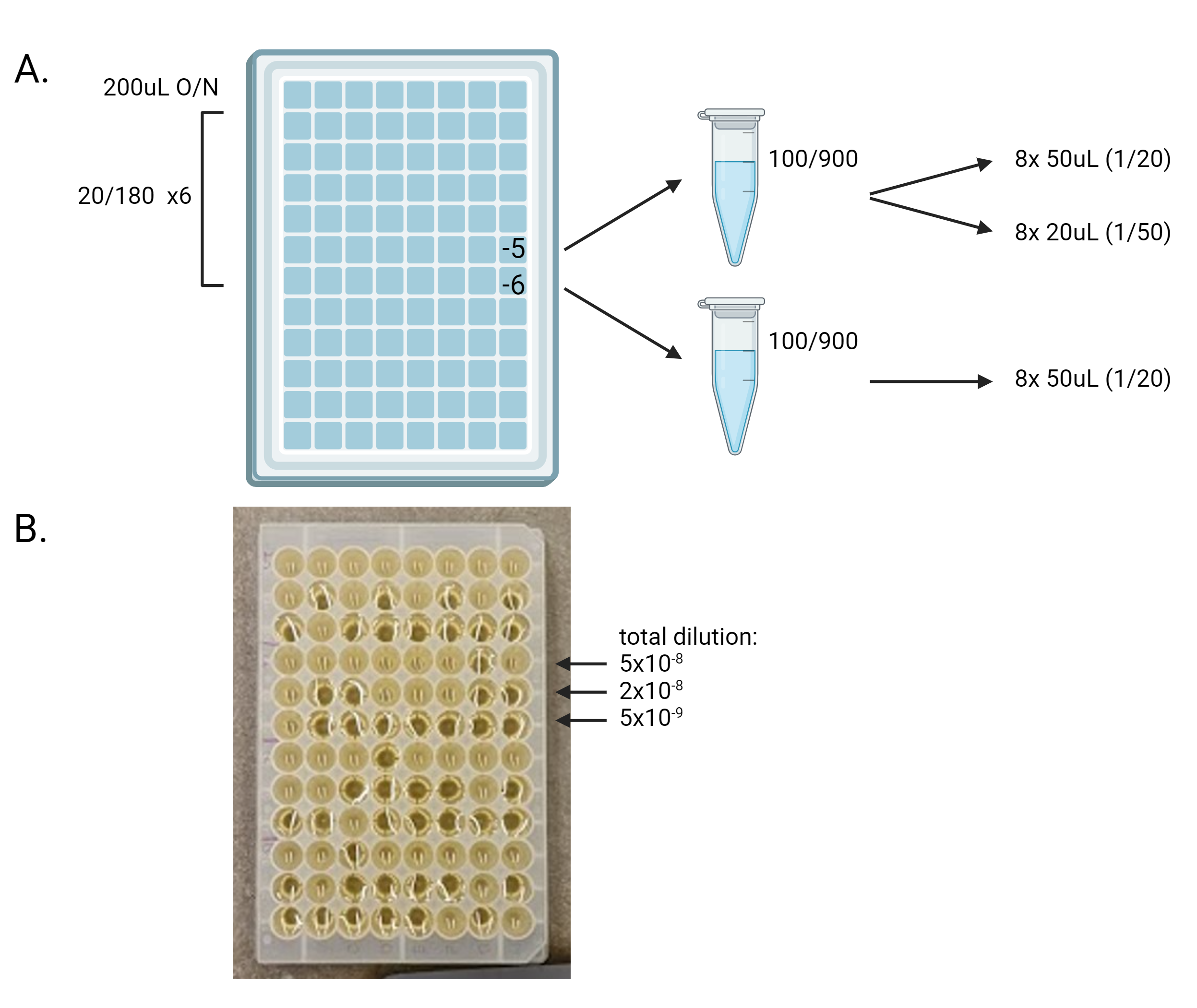

Supplement: evaf049_Supplementary_Data [file evaf049_supplementary_data.zip › Supplemental_serial_diilution_strategy_LvP.png]
